# Using Liposomes as Carriers for Polyphenolic Compounds: The Case of *Trans*-Resveratrol

**DOI:** 10.1371/journal.pone.0041438

**Published:** 2012-08-22

**Authors:** Claudia Bonechi, Silvia Martini, Laura Ciani, Stefania Lamponi, Herbert Rebmann, Claudio Rossi, Sandra Ristori

**Affiliations:** 1 Pharmaceutical and Applied Chemistry Departments & CSGI, University of Siena, Siena, Italy; 2 Chemistry Department “Ugo Schiff” & CSGI, University of Florence, Sesto Fiorentino, Italy; 3 Phospholipid Research Center, Heidelberg, Germany; University of Quebect at Trois-Rivieres, Canada

## Abstract

Resveratrol (3,5,4′-trihydroxy-*trans*-stilbene) is a polyphenol found in various plants, especially in the skin of red grapes. The effect of resveratrol on human health is the topic of numerous studies. In fact this molecule has shown anti-cancer, anti-inflammatory, blood-sugar-lowering ability and beneficial cardiovascular effects. However, for many polyphenol compounds of natural origin bioavailability is limited by low solubility in biological fluids, as well as by rapid metabolization *in vivo*. Therefore, appropriate carriers are required to obtain efficient therapeutics along with low administration doses.

Liposomes are excellent candidates for drug delivery purposes, due to their biocompatibility, wide choice of physico-chemical properties and easy preparation.

In this paper liposome formulations made by a saturated phosphatidyl-choline (DPPC) and cholesterol (or its positively charged derivative DC-CHOL) were chosen to optimize the loading of a rigid hydrophobic molecule such as resveratrol.

Plain and resveratrol loaded liposomes were characterized for size, surface charge and structural details by complementary techniques, i.e. Dynamic Light Scattering (DLS), Zeta potential and Small Angle X-ray Scattering (SAXS). Nuclear and Electron Spin magnetic resonances (NMR and ESR, respectively) were also used to gain information at the molecular scale.

The obtained results allowed to give an account of loaded liposomes in which resveratrol interacted with the bilayer, being more deeply inserted in cationic liposomes than in zwitterionic liposomes. Relevant properties such as the mean size and the presence of oligolamellar structures were influenced by the loading of RESV guest molecules.

The toxicity of all these systems was tested on stabilized cell lines (mouse fibroblast NIH-3T3 and human astrocytes U373-MG), showing that cell viability was not affected by the administration of liposomial resveratrol.

## Introduction

Polyphenols from plant extracts are molecules with recognized chemopreventive and therapeutic efficacy. Among them, resveratrol (3,5,4′-trihydroxy-*trans*-stilbene, henceforth also called RESV, Scheme S1), especially found in grapes and red wine, shows anti-microbial, anti-inflammatory, blood-sugar lowering ability, as well as beneficial cardiovascular effects [1], [2], [3]. In this context, resveratrol is pointed out as a possible contributor to the pharmacological protection against several human pathologies.

However, alike many polyphenolic compounds, RESV is characterized by poor bioavailability, weak absorption after oral administration and rapid metabolization *in vivo*. Therefore, high doses are required to reach significant beneficial effects. This is mainly due to limited solubility in aqueous media (<0.001 mol/l) and, consequently, in biological fluids. *Trans*-resveratrol is also converted into its cis isomer, a less active form, upon light exposure [4] [5]. Indeed, many studies have shown that the main antioxidant action is exerted by *trans*-resveratrol rather than *cis*-resveratrol. Examples concerning different cancer forms include the papers of Shu et al [6] [7], while Rius et al report on the superior anti-inflammatory activity of *trans*-resveratrol over *cis*-resveratrol [8].

A strategy to circumvent the above mentioned solubility limitations consists in loading polyphenols into water soluble carriers, which also offer chemical and biological protection.

Liposomes are biocompatible carriers that can be prepared from lipids with tunable physico-chemical properties and loaded with compounds of different lipophilic-hydrophilic nature [9] [10]. In particular, lipophilic drugs, such as resveratrol, are usually incorporated in the limiting bilayer [11]. Liposomes also enable slow release at the target site over prolonged periods of time.

Moreover, it has been shown that association with liposomes is an effective way to protect RESV from light and other degradative processes [12].

Previous studies constitute a good background for a detailed investigation of RESV insertion modality in liposomes with different properties [13] [14]. Indeed, this represents a critical issue to understand and optimize cell delivery.

In the present paper we designed and characterized liposome formulations based on rigid bilayers, in order to obtain good compatibility with the rigid aromatic portion of the polyphenol molecular structure. The liposome surface properties were varied from zwitterionic to cationic, since the outer charge of the carrier plays an important role in the first approach with cell membranes, driving the pathway for internalization.

DPPC (1,2-dipalmitoyl-*sn*-glycero-3-phosphocholine, Scheme S2a) was chosen as main bilayer component, due to the abundance of phosphocholine lipids in the plasma membrane of eukaryotic organisms. Cholesterol (5-cholesten-3ß-ol, henceforth called CHOL, Scheme S2b) and its cationic derivative DC-Chol (3ß-[N-(N′,N′-dimethylaminoethane)-carbamoyl]cholesterol, Scheme S2c) were also part of the liposome formulations, to improve the affinity toward raft domains in cell membranes and facilitate delivery.

Plain and resveratrol-loaded liposomes were extensively characterized by physico-chemical techniques, i.e. Dynamic Light Scattering (DLS), Zeta potential, Small Angle X-ray Scattering (SAXS), Nuclear Magnetic Resonance (NMR) and Electron Spin Resonance (ESR).

As a first step towards biological trials, toxicity was tested on different stabilized cell lines, such as mouse fibroblast NIH-3T3 and human Astrocytes U373-MG. Cell viability was demonstrated to be unaffected by the administration of plain and resveratrol loaded liposomes, thus ensuring the innocuous nature of all the systems here investigated.

## Materials and Methods

### Liposome Preparation

Resveratrol (3,5,4′-trihydroxy-*trans*-stilbene, ≥99% purity), cholesterol (5α-cholestan-3ß-ol ≥99.5 purity) and all solvents were purchased from Sigma. DPPC (1,2-dipalmitoyl-*sn*-glycero-3-phosphocholine, >99% purity) was purchased from Lipoid GmbH (Germany) and DC-Chol (3ß-[N-(N′,N′-dimethylaminoethane)-carbamoyl]cholesterol, >99% purity) was from Avanti Polar Lipids Inc., Alabaster, AL.

One liposomal formulation consisted of DPPC and Cholesterol at two different molar ratios: 50/50 and 75/25. A second liposomal formulation contained DPPC and DC-Chol at the same molar ratios.

Liposomes were prepared in a round bottom vial by mixing the appropriate amounts of stock solutions, which were 2^.^10^−2^ M in chloroform for lipids and 0.1 M in ethanol for resveratrol. A dry lipid film (with or without Resveratrol) was obtained by evaporating the solvent under vacuum overnight. Rehydrating with Milli-Q grade H_2_O (or D_2_O, for NMR experiments) yielded a mutilamellar dispersion. To obtain uniform liposomes with reduced or no lamellarity the following three steps were carried out: (i) homogenization by eight freeze-thaw cycles in liquid nitrogen and water bath at 50°C; (ii) sonication by five cycles of 20 s at 70% power level with a Bandelin Electronic Sonoplus HD2070 (Bandelin Electronic UW2070 tip) sonicator and (iii) extrusion through 100 nm polycarbonate membranes (27 passages) with a LiposoFast apparatus (Avestin, Ottawa, CA). All samples were submitted to sonication for consistency of preparation, though this step was not necessary in the case plain liposomes.

In all samples the total lipid concentration was 10^−2^ M

### UV quantification of trans-resveratrol on liposomes

UV-visible spectra were recorded at 25°C with a Perkin-Elmer Lamda 25 spectrophotometer. Quartz 10 mm path-length cuvettes were used.

Prior to spectra recording, liposome disruption was carried out in order get rid of the scattering background (scaling as λ^−4^), due to large aggregates in solution, which can affect precise intensity evaluation. To disrupt liposomes and release the entrapped RESV, samples were treated with sodium dodecyl sulfate in excess of ethanol and stored one hour at −30°C.

A calibration curve was built by measuring the absorbance of solutions with known resveratrol solution at 306 nm. This wavelength value was chosen to optimize detection of both RESV isomers [15].

### Size and surface charge of liposomes

The size and surface charge of plain and RESV loaded liposomes were measured by. Dynamic Light Scattering, DLS, (Coulter Sub-Micron Particle Analyzer N4SD, equipped with a 4 mW helium-neon laser and 90° detector) and Zeta potential (Coulter DELSA 440 SX), respectively. In particular, the autocorrelation function of the scattered light was analyzed by the cumulant method [16] to obtain the mean size and polydispersity index (P.I.), while Zeta potential values were calculated from the electrophoretic mobility by means of the Helmholtz-Smoluchowski relationship [17].

### Small Angle X-ray Scattering experiments

SAXS patterns, consisting of the scattered intensity I as a function of the moment transfer q = (4π/λ) sinθ (where 2θ is the scattering angle), were recorded at the high brilliance ID02 beamline of the ESRF (European Synchrotron Radiation Facility, Grenoble, France). The q-range covered was 0.103–4.887 nm^−1^. Samples were placed in 1.5 mm diameter glass capillaries and at least 3 curves were recorded at different points along each capillary.

The measured SAXS diagrams were normalized to an absolute scale using standard procedure [18]. Fitting of SAXS profiles was performed with the Global Analysis Program (GAP), provided by Dr Georg Pabst (http://www.ibn.oeaw.ac.at/people/Georg/index.html), which allows to reproduce the SAXS pattern of bilayer-based strctures, i.e. vesicles and lamellar phases, by using the following equation:
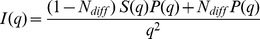
(1)where N_diff_ is the fraction number of uncorreraled bilayers per scattering domain, S(q) is the structure factor describing inter-aggregate interactions and P(q) is the the absolute square of a bilayer form factor. The contribution of oligolamellar structures was evidenced in the shape of SAXS profiles by the appearance of Bragg peaks with q spacing n^.^2π/d, where n is an integer number giving the order of the peak and d is the repeating distance for the bilayer plus the intercalated water. In this case, a structure factor based on the Caille' theory [19] was chosen for the fitting. This model includes a fluctuation parameter η accounting for the tendency of single bilayers to create local non zero curvature regions.

It is to be noted that in the case of diluted systems, such as the solutions of non interacting (i.e. stable) liposomes, laboratory SAXS apparatii do not usually provide enough resolution and good signal to noise ratios to allow a detailed interpretation of the intensity profiles.

### Nuclear Magnetic Resonance experiments

NMR experiments were performed using a Bruker DRX-600 Avance spectrometer operating at 600.13 MHz for ^1^H, equipped with an xyz gradient unit. All samples were prepared in D_2_O.

Spectra were processed on Silicon Graphics workstations by Bruker XWinNMR software (version 2.5) and NMRPipe software [20].

Two dimensional spectra (NOESY and dqf-COSY) were acquired with 2048 complex points for 512 experiments. NOESY spectra were acquired with 10 s recycle delay, TPPI phase cycling and mixing time of 200 and 400 ms.

### Electron Spin Resonance experiments

ESR spectra were recorded with a Bruker ESR spectrometer model 200D, working in the continuous wave mode at X-band (9.5 GHz). Samples were inserted in the typical rectangular cavity. Data acquisition and handling were carried out with the ESR software commercialized by STELAR (Meda, Italy). Temperature was controlled with the Bruker VT 3000 apparatus (accuracy (0.5°C).

5, 12 and 16 doxyl-stearic acid spin probes (5-DSA, 12 DSA and 16-DSA, respectively) were purchased from Sigma Chemicals, München, Germany, and used as received.

The right amount of stock ethanol solutions of spin probes were added to the CHCl_3_ solution of lipids in molar ratio 1∶100. This way the nitroxides were easily intercalated into the bilayer, and gave information about the hydrophobic region of plain and resveratrol laded liposomes, as described in the text.

### Cell culture

Mouse tumoral fibroblasts NIH3T3 (Abcam, Cambridge, UK) and human astrocytes U3763-MG (American Type Culture Collection, USA) were used for citotoxicity experiments. Dulbecco's Modified Eagle's Medium (DMEM) was from Lonza (Belgium), and all other chemicals were purchased from Sigma-Aldrich (Germany).

Cells were propagated in DMEM with 10% fetal calf serum, 1% L-glutamine-penicillin-streptomycin solution, and 1% MEM Non-Essential Amino Acid Solution, at 37°C in a humidified atmosphere containing 5% CO_2_.

Once cells reached the 50% of confluence (i.e. after 24 hrs of culture) the medium was discharged and liposomes, properly diluted in complete medium, were added to each well. A concentration of plain and RSV loaded liposomes 1% v/v was tested. All samples were set up in six replicates. After 24 h of incubation, cell morphology was evaluated by optical microscopy (Olympus BX40) and cell viability was checked by Neutral Red uptake (Sigma-Aldrich, Switzerland) using the procedure reported in a previous paper [21].

For the statistical analysis, multiple comparison were performed by one-way ANOVA and individual differences tested by Fisher's test after the demonstration of significant intergroup differences by ANOVA. Differences with p<0.05 were considered significant.

## Results and Discussion

The preparation procedure followed in this work allowed to obtain good incorporation rates, that is over 50% of the initial RESV used in the preparation. Results are reported in tables 1 and 2, for zwitterionic and cationic liposomes, respectively, together with the corresponding mean size and polydispersity index P.I., measured by DLS, and Zeta potential values.

**Table 1:** Characteristics of DPPC/CHOL (zwitterionic) liposomes.

Lipid ratio	Initial RESV concentration (M)	Loaded RESV (M)	Mean diameter (nm)	P.I.	Zeta potential (mV)
50/50	0	-	240±30	0.56	−10±3
50/50	1.25 10^−3^ M	5.5 10^−4^ M	370±30	0.57	−6±2
75/25	0	-	250±40	0.61	−6±3
75/25	1.25 10^−3^ M	6.9 10^−4^ M	360±20	0.42	−1±4

**Table 2:** Characteristics of DPPC/DC-CHOL (cationic) liposomes.

Lipid ratio	Initial RESV concentration (M)	Loaded RESV (M)	Mean diameter (nm)	P.I.	Zeta potential (mV)
50/50	0	-	130±15	0.31	+54±8
50/50	1.25 10^−3^ M	5.9 10^−4^ M	110±15	0.32	+57±8
75/25	0	-	110±15	0.33	+57±8
75/25	1.25 10^−3^ M	5.4 10^−4^ M	110±10	0.28	+56±8

Analyzing the Zeta Potential values in Table 1 we observe that plain DPPC/CHOL liposomes have a small negative surface charge, though the net polar head charge of zwitterionic phospholipids is zero. Indeed, it has been reported that liposomes made by PC components behave as slightly negative aggregates when in the presence of an external electric field [22]. This may be due to preferential absorption of HO^−^ ions from the water environment or to the outward exposure of phosphate groups. Upon addition of resveratrol the surface charge of DPPC/CHOL liposomes did not change markedly; on the contrary, their mean size was much larger.

For cationic liposomes no appreciable variation of the mean diameter was observed when resveratrol was associated to liposomes and also the surface charge didn't change its high positive value.

**Figure 1:**
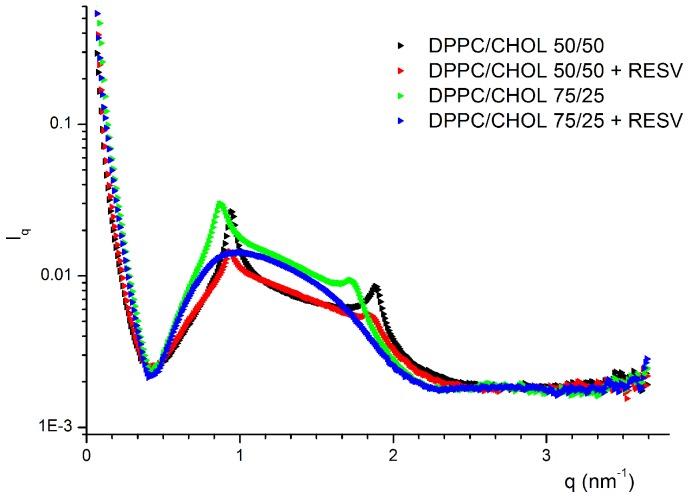
SAXS diagrams of plain and resveratrol loaded zwitterionic liposome.

The SAXS intensity diagrams of DPPC/CHOL plain and resveratrol loaded liposomes are shown in Figure 1.

**Figure 2:**
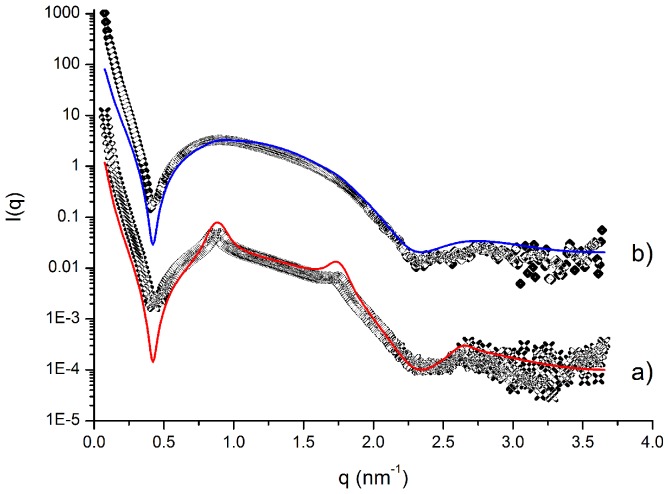
Experimental SAXS diagrams (symbols) and fitting (continuous lines) of DPPC/CHOL 75/25 plain and RESV loaded liposomes (a and b, respectively). The best fit parameters used are listed in table 3. The curves of RESV loaded liposomes have been vertically shifted for the sake of clarity.

Fitting with the Global Analysis Program showed that these intensity profiles were given by the superposition of two signals: (i) a diffuse scattering, represented by the large bump in the range q ∼ 0.4–2 nm^−1^, which is typical of monolamellar vesicles and (ii) the sequence of two Bragg peaks with q_max_ (II order)∶ q_max_ (I order) = 2∶ 1, arising from stacked bilayers, that is from oligolamellar vesicles. The relevant best fit parameters obtained for zwitterionic liposomes are listed in table 3 and two examples of fitting are shown in figure 2.

**Table 3:** Best fit parameters for plain and resveratrol loaded DPPC/CHOL liposomes.

	DPPC/CHOL 75/25	DPPC/CHOL 75/25+RESV	DPPC/CHOL 50/50	DPPC/CHOL 50/50+RESV
d	71.48	71.48	66.3	67
Nlam	3	2.3	2.8	2.6
η	0.05	0.05	0.1	0.1
Ndiff	0.85	0.99	0.88	0.94
Bilayer thickness (Å)	68	68	60	60

From Table 3 we observe that DPPC-CHOL liposomes contained a small but significant fraction of oligolamellar structures (2–3 stacked bilayers), which decreased upon Resveratrol loading. This indicated that RESV was located on the surface of these liposomes and that its presence modified some bilayer properties, such as the overall curvature, thus increasing the liposome size, as it was also revealed by the DLS results reported in table 1. Otherwise, Resveratrol at this moderate content (with respect to total lipids) did not significantly affect either the bilayer rigidity (η) or its thickness. On the other hand, these latter parameters underwent appreciable variation with the relative molar ratio of DPPC and CHOL, showing that increasing cholesterol amount rendered the bilayer more fluid and less thick.

**Figure 3:**
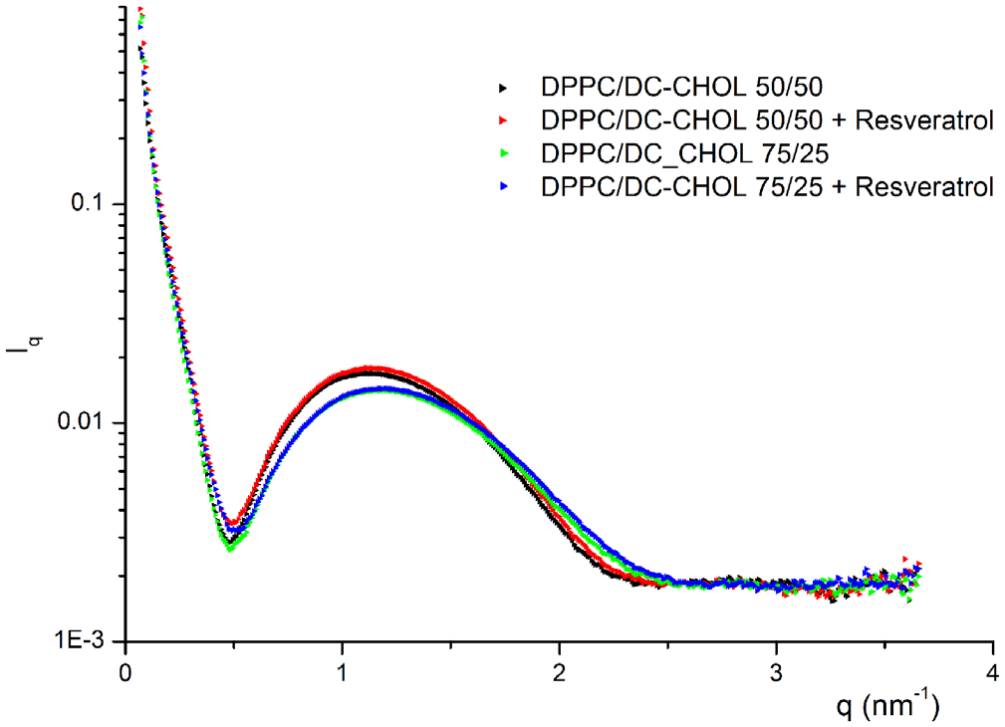
SAXS diagrams of plain and resveratrol loaded cationic liposomes.

The SAXS diagrams of DPPC/DC-CHOL liposomes (Figure 3) demonstrated that these liposomes were entirely monolamellar and that their structure was retained upon RESV loading. This finding was in agreement with the fact that the mean diameter of DPPC/DC-CHOL liposomes measured by DLS (table 2) was very close to the pore size used for extrusion and it didn't change after resveratrol association. The best fit parameters obtained for plain and resveratrol loaded DPPC/DC-CHOL liposomes are reported in Table 4.

**Table 4:** Best fit parameters for plain and resveratrol loaded DPPC/DC-CHOL liposomes.

	DPPC/DC-CHOL 75/25	DPPC/DC-CHOL 75/25+RESV	DPPC/DC-CHOL 50/50	DPPC/DC-CHOL 50/50+RESV
d	n.a.	n.a.	n.a.	n.a.
Nlam	1	1	1	1
η	n.a.	n.a.	n.a.	n.a.
Ndiff	1	1	1	1
Bilayer thickness (Å)	71	70	63.6	63.6

More details on the association modality between resveratrol and the liposome bilayers were given by Magnetic Nuclear and Electron Spin Resonances.

**Figure 4:**
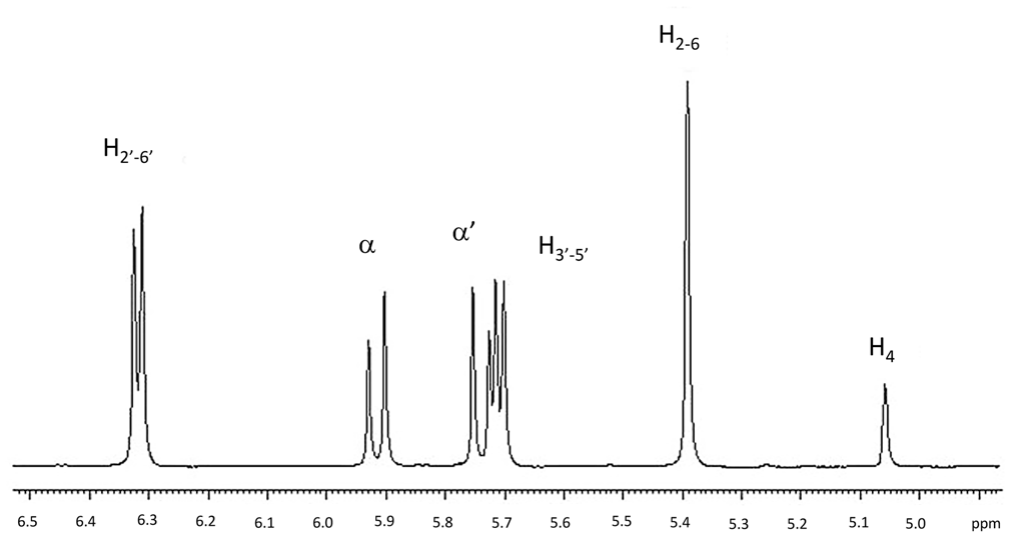
^1^H spectrum of *trans*-Resveratrol in D_2_O/DMSO (2/3) recorded at 600 MHz and 298 K.

The ^1^H monodimensional NMR spectrum of *trans*-resveratrol is reported in figure 4, together with the corresponding hydrogen numbering and assignment [23]. This spectrum was recorded in a mixture D_2_O/DMSO = 2/3, due to the scarce solubility of resveratrol in water.

**Figure 5:**
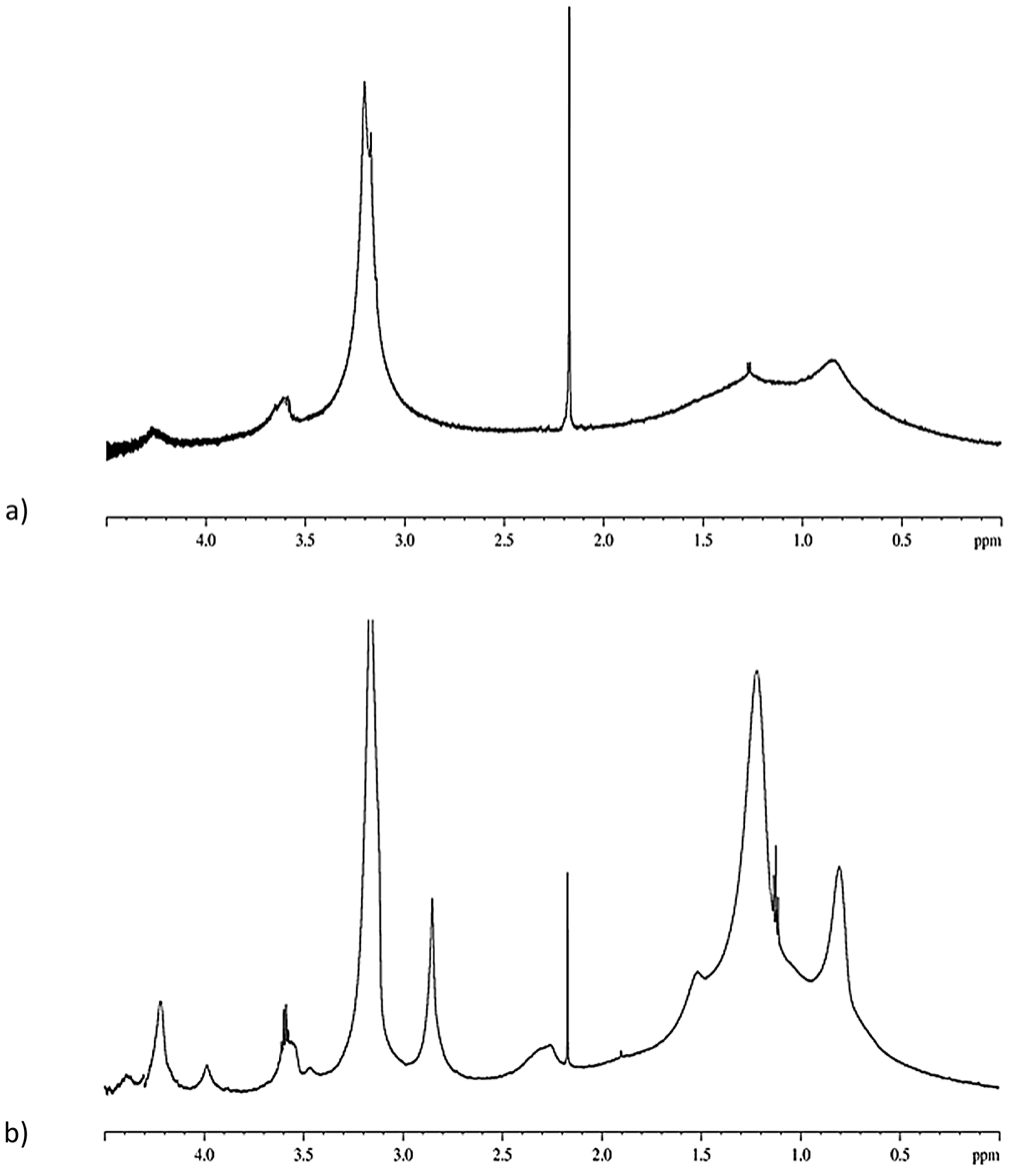
NMR proton spectrum in the range 0–4.5 ppm for: a) DPPC/Chol liposome in D_2_O and b) DPPC/DC-CHOL liposome in D_2_O.

The ^1^H-NMR spectra of DPPC/CHOL and DPPC/DC-CHOL liposomes are shown in Figure 5a and 5b, respectively. Both these spectra showed broad unresolved peaks, which are typical of macromolecule or aggregates. In particular, liposome reorientational slow motions did not allow to mediate the interactions between the protons of lipid molecules packed in the bilayer, where they experienced a remarkable degree of order. The broad signals and the resolution obtained in liposomes didn't also allow the identification of proton-proton scalar couplings. It is to be noted that proton peaks were more resolved in cationic liposomes than in zwitterionic liposomes, in agreement with the smaller size of the former aggregates (see table 1 and table 2).

The assignment of observable signals allowed to identify the methyl and methylenic protons lipid chains as resonating in the region 0.85–1.50 ppm. The –CH_3_ groups of the DPPC polar heads resonated at 3.2 ppm, while the methylenic protons (6 and 9) of choline were found at 3.6 ppm and 7,8, 10 protons resonated at about 4.3 ppm [24].

The signal at 2.85 ppm, which was not present in the spectrum of DPPC/CHOL liposomes, was attributed to the methylenic protons of the DC-CHOL polar head.

**Figure 6:**
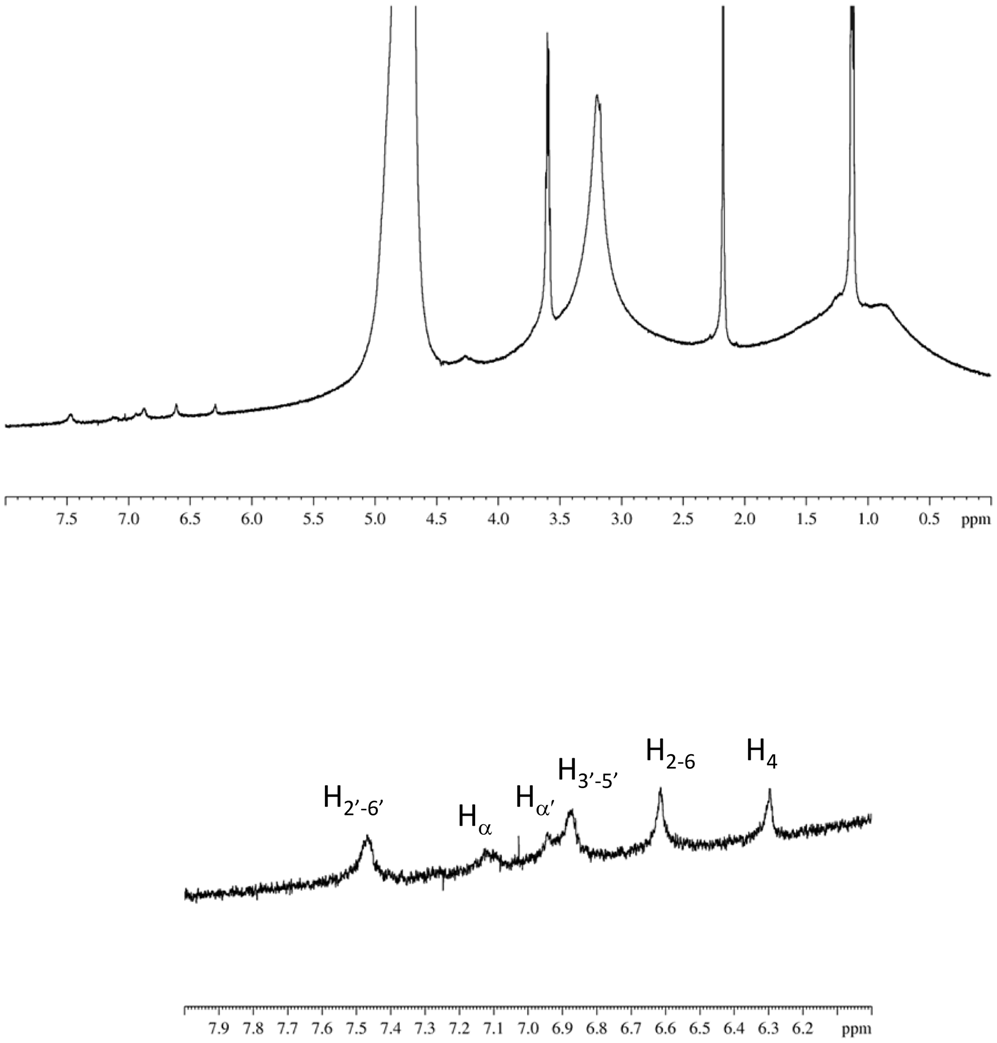
^1^H NMR spectrum of zwitterionic liposomes and corresponding enlargement of the aromatic region.

The NMR spectra of pure liposomes did not show any detectable signal in the aromatic region (6–8 ppm). On the contrary, when RESV was loaded into DPPC-CHOL liposomes weak signals could be detected in this region. This behavior is exemplified in enlargement reported in figure 6.

**Figure 7:**
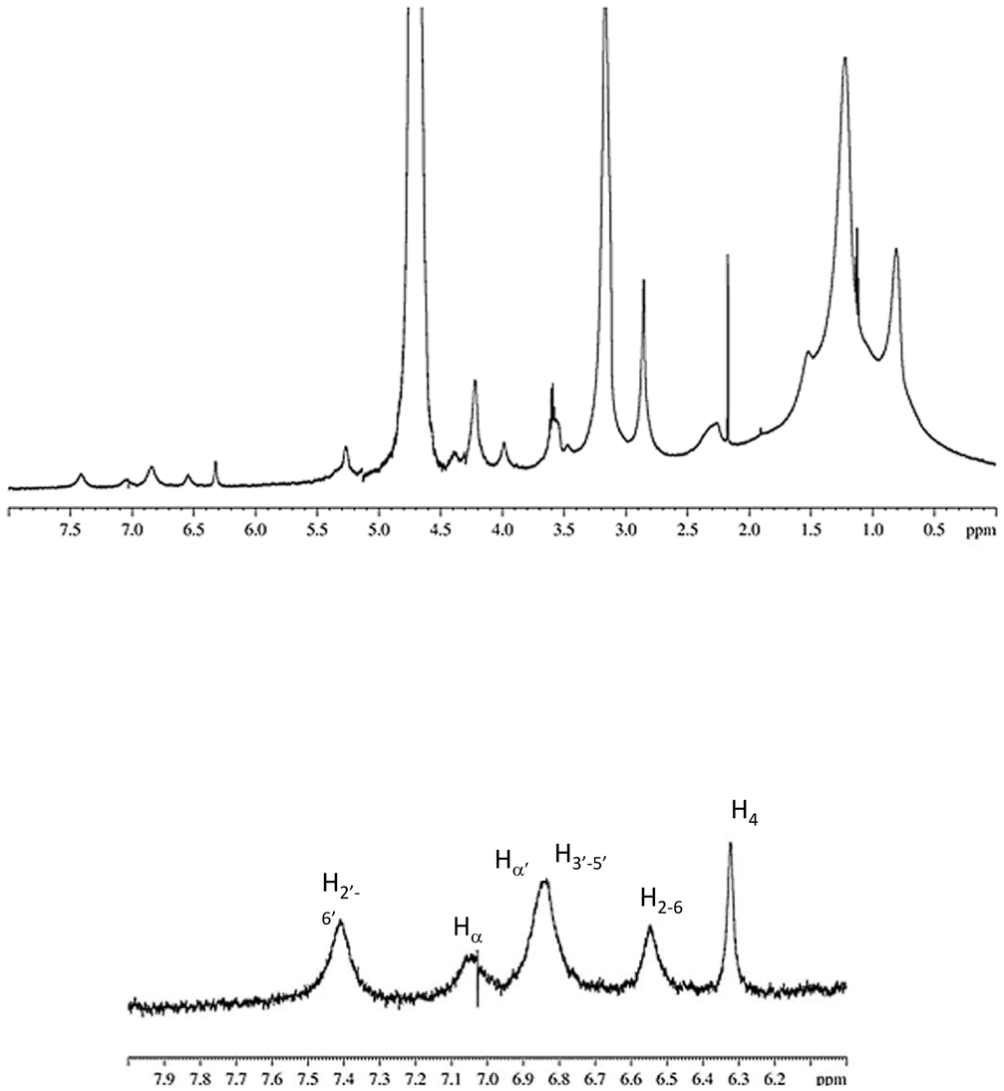
^1^H NMR spectrum of cationic liposomes and corresponding enlargement in the aromatic region.

At first sight, the case of resveratrol loaded DPPC/DC-CHOL liposomes was similar to DPPC/CHOL liposomes, as it can be inferred from the spectrum shown in Figure 7.

However, the signal observed at 5.2 ppm was identified as due to the proton 6 of the DC-CHOL molecule, suggesting a different location of resveratrol in the bilayer of cationic liposomes with respect to zwitterionic liposomes. This hypothesis was confirmed by the two dimensional (NOESY) spectrum of DPPC/DC-CHOL liposomes loaded with resveratrol.

**Figure 8:**
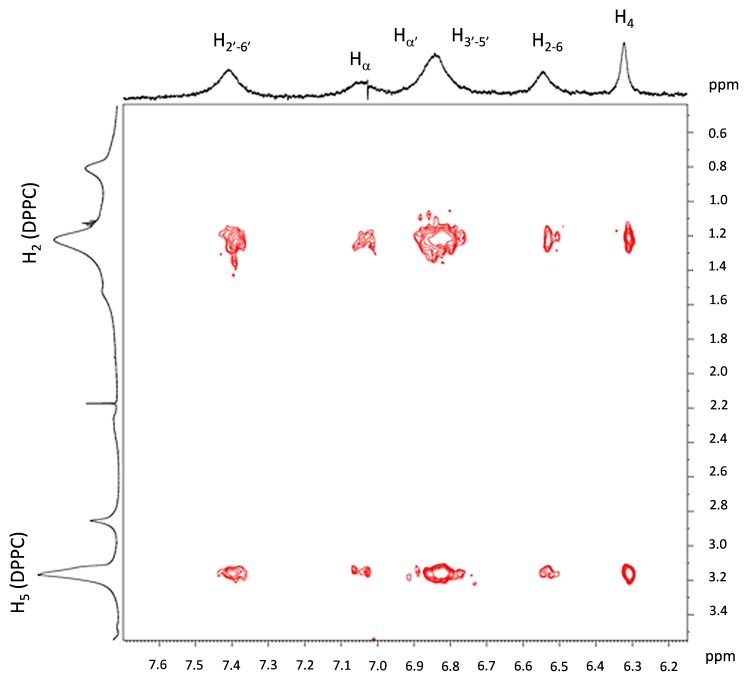
NOESY spectrum of DPPC/DC-CHOL RESV loaded liposomes in D_2_O recorded at 600 MHz and 298 K.

NOESY experiments are able to evidence the spatial separation of protons up to a distance of 4.5 Å, since for such neighbor atoms cross peaks are detectable. The enlargement of NOESY spectrum reported in figure 8 demonstrated that resveratrol hydrogens were very close to the hydrogens of the lipid chains in cationic liposomes. In particular, dipolar couplings between all protons of resveratrol with the -CH_2_ (2) of lipid chain and with the -CH_3_ protons of the DPPC polar head were evident, indicating that resveratrol was inserted deep inside the bilayer of cationic liposomes. This was in perfected agreement with the SAXS results, as discussed above.

**Figure 9:**
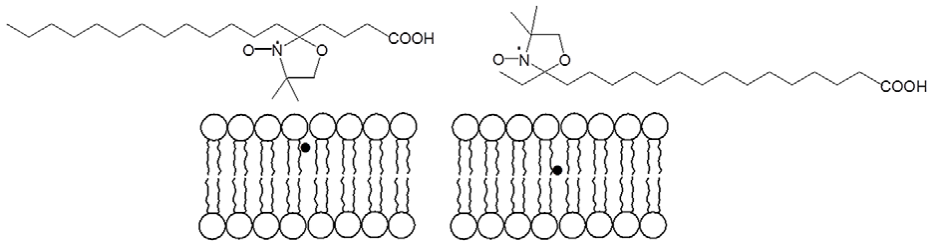
Sketch of the interbilayer location of the 5-DSA (left) and 16-DSA (right) spin probes.

Further information on the molecular packing of liposome bilayers was given by ESR. This technique makes use of paramagnetic probes when studying systems without unpaired electrons. In particular, to study the molecular ordering and fluidity in lipid bilayers, nitroxide derivatives of n-doxylstearic acid (n-DSA) are valuable tools [25] [26]. n-DSA probes are amphiphile molecules themselves, and possess a paramagnetic unit, i.e. the nitroxide group, on the n*th* carbon atom of the alkyl chain, that is at different distance from the polar head (COOH). Therefore, n-DSAs are able to report on different regions of the lipid bilayer, as shown in Figure 9 for the examples of 5-DSA and 16-DSA.

**Figure 10:**
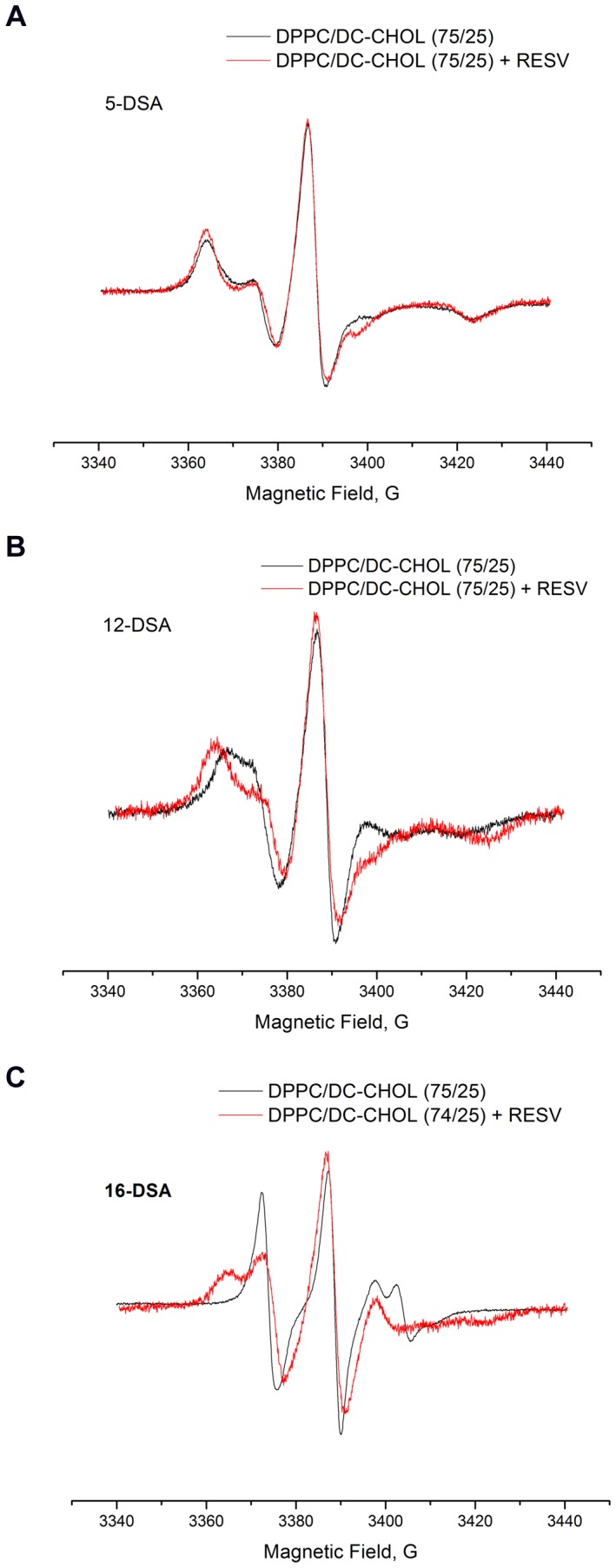
ESR spectra of 5-DSA, 12-DSA and 16-DSA (a,b and c, respectively) inserted in the bilayer of plain and RESV loaded cationic liposomes. The three probes have their paragnetic unit located progressively deeper in the hydrocarbon region.

In the case of cationic liposomes the ESR spectra showed that no significant difference was sensed by the 5-DSA probe (Figure 10a) in plain and RESV loaded liposomes, whereas marked changes took place in the central part of the lipid bilayer upon resveratrol association, as evidenced by the spectra of 12-DSA and 16-DSA (figure 10b and 10c, respectively).

This finding was in excellent agreement with the hypothesis of resveratrol location in the inner part of cationic liposome bilayers, as indicated by SAXS and NMR data.

Moreover, the ESR spectra of nitroxide spin probes showed that no marked variations could be evidenced within the bilayer of zwitterionic liposomes after loading with resveratrol (data not shown). This meant that resveratrol was associated with the liposome surface and did not penetrate significantly in the hydrocarbon region.

**Figure 11:**
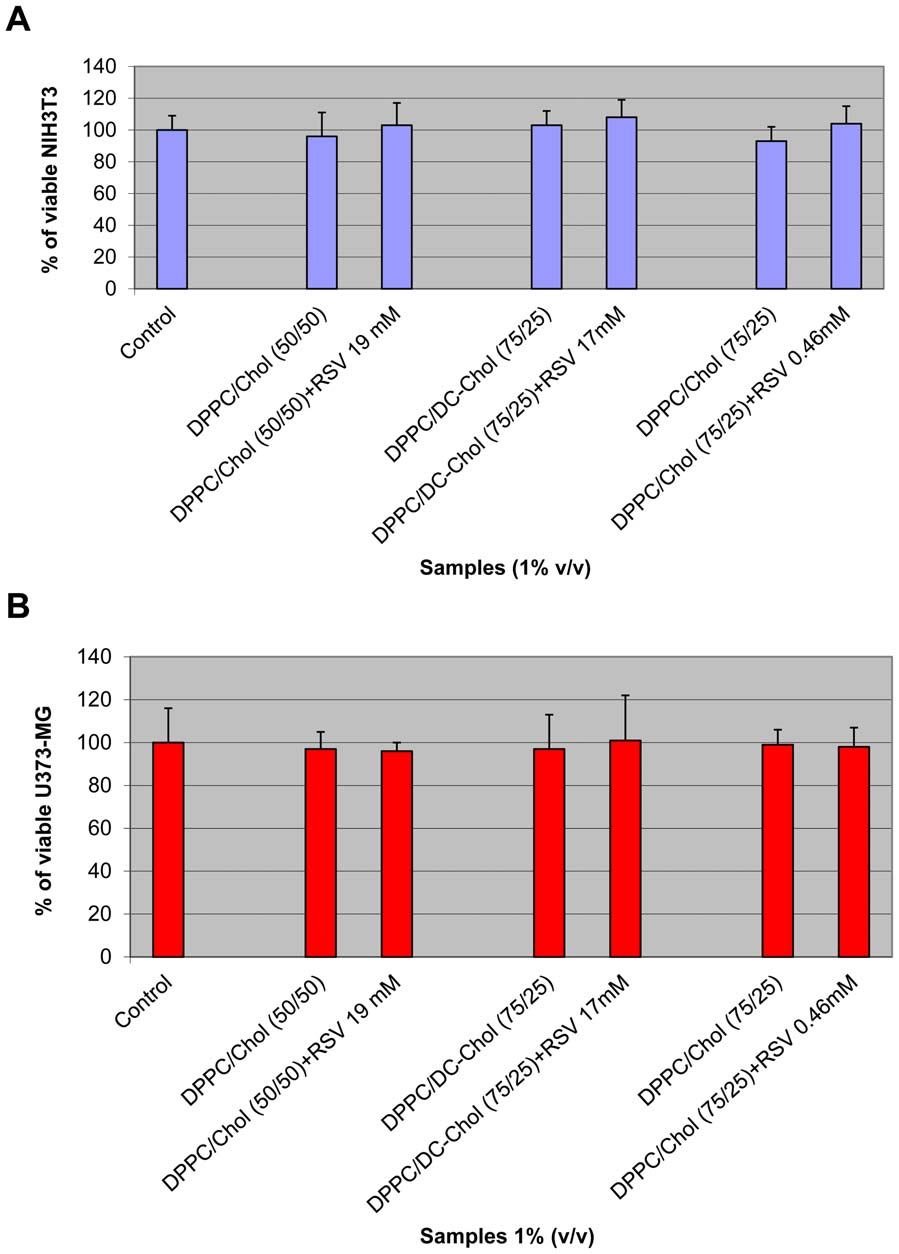
Percentage of viable NIH3T3 (a) and U373-MG (b) after 24 h of contact with plain and RESV loaded liposomes, as determined by the Neutral Red Uptake. Data are the mean ± SD of three experiments run in six replicates. No value was statistically different versus control (complete medium).

Toxicity experiments represent a necessary step before using drug and drug carriers in biological applications. Here cell administration of plain and RESV loaded liposomes was performed to NIH3T3 and U3763-MG (mouse fibroblasts and astrocytes) stabilized cell cultures. None of the investigated systems showed marked toxicity level toward both kinds of cells, as demonstrated by viability data obtained Neutral Red uptake (figures 11a and 11b). In fact, optical microscopy analysis showed that after 24 hour treatment NIH3T3 and U373-MG had excellent spreading and maintained the characteristic morphology of spindle shaped cells and “islands” distribution, respectively (data not shown). The adhesion of cells to the polystyrene was identical on each sample and no evidence of cytotoxicity such as cell lysis was found.

## Conclusions

In this work we studied the association of trans-Resveratrol with different formulations of zwitterionic and cationic liposomes, which were designed *ad hoc* to optimize RESV loading.

Rigid bilayers made by DPPC were chosen to obtain good compatibility with substantially rigid guest molecules. Cholesterol and its positively charged derivative DC-CHOL were included in the formulation to improve cell delivery by enhancing the interaction with plasma membranes.

**Figure 12:**
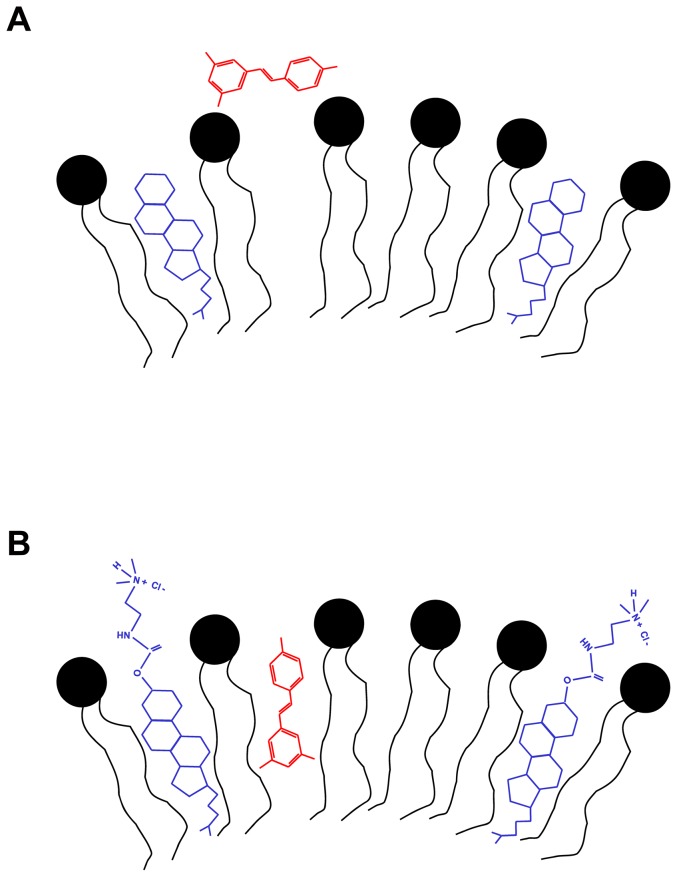
Sketch of RESV insertion in the bilayer of DPPC/CHOL (a) and DPPC/DC-CHOL(b) liposomes.

Extensive physico-chemical characterization allowed to draw a model in which size, surface charge, lamellarity and resveratrol location were assessed. In particular, the analysis and comparison of DLS, SAXS, NMR and ESR results pointed out marked differences in RESV location within the bilayer of zwitterionic and cationic liposomes, as it is shown in Figures 12a and 12b, respectively.

Liposome overall size and lamellarity, which are known to affect delivery efficacy, protection against biological degradation and metabolization time, were also evaluated.

Finally, as a first step towards biological trials, the innocuous nature of all the investigated systems was checked by administration to NIH3T3 and U373-MG stabilized cell lines.

We believe that the information obtained in this study are an important pre-requisite to prepare systems with defined properties for Resveratrol administration *in vitro* and *in vivo*.

## Supporting Information

Scheme S1 Structure and atomic numbering of trans-resveratrol (RESV).
(TIF)

Scheme S2 Molecular structure and atomic numbering of 1,2-dipalmitoyl-sn-glycero-3-phosphocholine (DPPC, a); Cholesterol (CHOL, b) and 3ß-[N-(N′,N′-dimethylaminoethane)-carbamoyl]cholesterol (DC-CHOL, c).
(TIF)
